# Application of Orthoflavivirus Pseudovirus Technology in Antiviral Research

**DOI:** 10.3390/ijms27020722

**Published:** 2026-01-10

**Authors:** Yalan Zhang, Yaqi Zhao, Chaojun Wang, Yuanyuan Zhou, Hao Yuan, Xiaodan Li, Yong Wang, Xiaoling Pan

**Affiliations:** 1 Department of Medical Laboratory Science, School of Medical Technology and Translational Medicine, Hunan Normal University Health Science Center, 371 Tongzipo Road, Changsha 410013, China; 202320193532@hunnu.edu.cn (Y.Z.); 202420193656@hunnu.edu.cn (Y.Z.); 16846@hunnu.edu.cn (C.W.); 202570193937@hunnu.edu.cn (Y.Z.); 201630192023@hunnu.edu.cn (H.Y.); lxd@live.cn (X.L.); 2 Department of Forensic Science, School of Basic Medical Sciences, Central South University, Changsha 410013, China; wangyong@csu.edu.cn

**Keywords:** orthoflavivirus, pseudovirus, neutralization assay, antiviral screening, vaccine evaluation

## Abstract

Arthropod-borne orthoflaviviruses, including dengue, Zika, Japanese encephalitis, yellow fever and West Nile viruses, pose a significant global public health threat, causing hundreds of millions of infections annually with severe clinical symptoms. However, the lack of effective vaccines and antiviral drugs, coupled with the biosafety risks associated with handling live highly pathogenic strains, hinders progress in antiviral research. Pseudovirus technology, which uses single-round infectious viral particles lacking replication competence, has thus gained prominence as a safe and versatile tool for antiviral research. This review systematically summarizes the construction, optimization, and applications of orthoflavivirus pseudoviruses in antiviral research. The primary construction strategies of orthoflavivirus pseudoviruses rely on multi-plasmid co-transfection of viral replicons and structural protein expression vectors, leveraging the host cell secretory pathway to mimic natural viral assembly and maturation. The core applications of pseudovirus technology are highlighted, including high-throughput screening and detection of neutralizing antibodies, identification of antiviral drugs targeting viral entry or replication, and evaluation of vaccine immunogenicity. Despite these strengths, the approach still faces limitations, such as incomplete simulation of native viral structures and batch-to-batch titer variability, which may affect the physiological relevance of findings. In summary, orthoflavivirus pseudovirus technology has become an essential platform in both basic virology research and translational medicine, providing critical insights and tools in the ongoing fight against arthropod-borne orthoflaviviruses diseases.

## 1. Introduction

Over the past decades, substantial efforts and resources have been dedicated to controlling diseases caused by arthropod-borne orthoflaviviruses, a group of enveloped, positive-sense, single-stranded RNA viruses belonging to the Flaviviridae family that includes prominent human pathogens such as Dengue virus (DENV), Zika virus (ZIKV), Japanese encephalitis virus (JEV), Yellow fever virus (YFV), and West Nile virus (WNV) [1,2]. Collectively, these viruses account for approximately 400 million infections and 100 million symptomatic cases globally each year [3]. Human infections with orthoflaviviruses can lead to severe outcomes, including hemorrhagic fever, serious neurological complications, and fatal diseases, thereby posing a significant threat to public health [4,5]. Recently, warming climate trends, ongoing urbanization, and the expanding distribution of arthropod vectors have jointly promoted the increasing prevalence of orthoflaviviruses, particularly in tropical and subtropical regions [6]. This emerging trend has heightened the global disease burden and exacerbated public health challenges. Given the lack of effective antiviral drugs and the absence of broad-spectrum vaccines for most orthoflavivirus infections [7,8], there is an urgent need to develop effective control strategies for orthoflavivirus. To achieve this goal, key challenges in orthoflavivirus control have to be addressed, including how to handle highly pathogenic orthoflavivirus when high-biosafety level facilities are limited, and how to establish efficient screening platforms for antiviral molecule discovery.

Given the limited availability of high-biosafety level facilities, pseudoviruses have achieved prominent progress in research on antibodies and antiviral drugs targeting live highly pathogenic viruses such as SARS-CoV-2 [9,10]. As replication-defective recombinant viral particles, they possess only single-round infectivity, meaning they do not propagate new viral progeny after infecting host cells [11]. This unique characteristic allows for safe manipulation with reduced biosafety risks [11]. Notably, pseudoviruses enable the safe and efficient simulation of viral entry mechanisms, and are widely applied in research fields such as neutralizing antibody evaluation, viral receptor identification, and antiviral drug screening [12]. With the development of molecular biology technology, pseudoviruses have demonstrated increasingly critical application value in responding to emerging flavivirus outbreaks and conducting research on variant strains [13]. Owing to their safety, operability and functional fidelity, pseudovirus technology has significantly promoted the basic research of flaviviruses and the development of antiviral strategies, providing key technical support for addressing such global public health threats.

The present review aims to investigate the construction and optimization strategies of orthoflavivirus pseudovirus, and provide a comprehensive overview of their applications in detection of neutralizing antibodies, evaluation of antiviral drugs, and vaccine development. Furthermore, the latest cutting-edge advances and challenges of orthoflavivirus pseudovirus technology in antiviral research are discussed.

## 2. Generation of Orthoflavivirus Pseudovirus

### 2.1. Construction Strategy of Orthoflavivirus Pseudovirus

The selection of an appropriate packaging system is the critical step in pseudovirus construction. Currently, the most widely used systems are based on either the vesicular stomatitis virus glycoprotein-deleted vector (VSVΔG) or lentiviral vector, each with distinct strength. By replacing the VSV-G protein with the envelope protein of a target virus (e.g., prM-E of flaviviruses), the VSVΔG system allows efficient production of high-titer pseudoviral particles [14,15,16]. Owing to its robust ability to activate innate immunity, this system exhibits unique advantages in vaccine immunogenicity research, as it typically elicits potent humoral and cellular immune responses [17]. In contrast, the lentiviral vector system is more extensively employed in investigations into viral entry mechanisms, receptor identification, and experimental settings requiring long-term reporter gene expression. This is attributable to its ability to integrate into the host genome for stable transduction and its high compatibility with heterologous envelope proteins during packaging process [18,19]. Nevertheless, both systems are subject to packaging constraints. The RNA packaging efficiency of lentiviral vector systems is strictly regulated by the interaction between Gag protein and the packaging signal. While achieving an optimal balance between helper plasmids and packaging plasmids is essential during production for VSVΔG system to avoid reduced packaging efficiency due to improper structural proteins expression [20,21,22]. Notably, particularly for orthoflavivirus pseudovirus construction, the orthoflavivirus backbone-based pseudovirus construction strategy achieves improved safety compared with the lentiviral vector-based pseudovirus construction strategy, owing to its independence from gene integration. In contrast to the VSVΔG -based pseudovirus construction strategy, the orthoflavivirus backbone-based pseudovirus construction strategy exhibits greater similarity to native viruses, with an immune activation capacity more comparable to that of native orthoflavivirus, attributable to its homologous mimicry of the orthoflavivirus capsid and core expression and structures [17,18,22].

**Figure 1:**
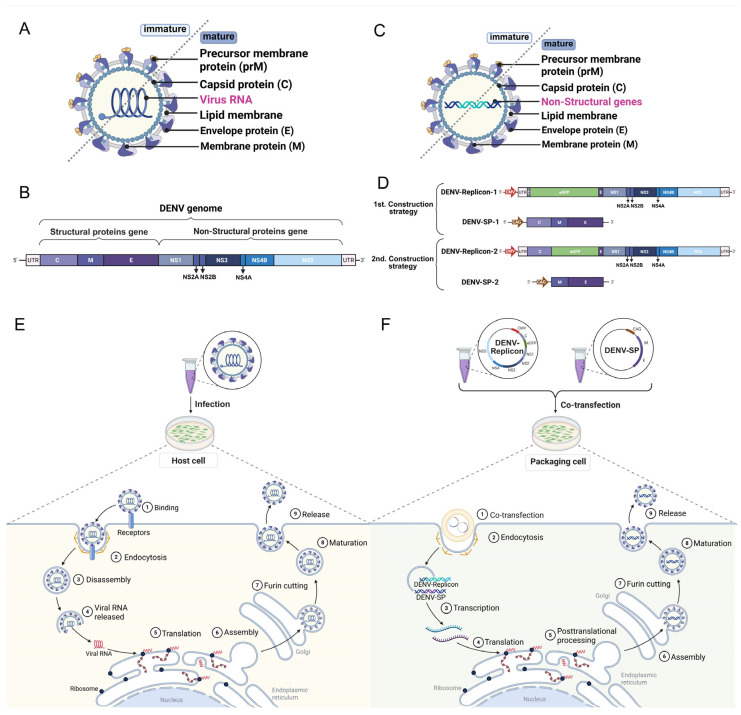
Schematic diagram of dengue virus (DENV) replication and orthoflavivirus pseudovirus generation. (**A**) Schematic diagram of DENV particle; (**B**) DENV genome; (**C**) Schematic diagram of DENV pseudovirus particle; (**D**) DENV replicon and structural protein plasmids; (**E**) DENV life cycle: ① Virus binds to host cell receptors; ② Virus enters cells via receptor-mediated endocytosis; ③ Virus disassembly; ④ Viral RNA is released into the cytoplasm; ⑤ Ribosomes translate viral RNA, Structural protein precursor (prM/E) is synthesized and modified in the endoplasmic reticulum (ER); ⑥ Viral assembly; ⑦ prM protein is cleaved by furin protease to form mature M protein; ⑧ Virus maturation; ⑨ Mature virus particles are released from the cell via budding; (**F**) Orthoflavivirus pseudoviruses generation process: ① Co-transfection of plasmids; ② Plasmids enter cells via endocytosis; ③ Plasmid DNA is transcribed into mRNA; ④ Ribosomes translate replicons and structural proteins separately; ⑤ Structural prM/E is synthesized and modified in the ER; ⑥ Pseudovirus assembly; ⑦ prM protein is cleaved by furin protease to form mature M protein; ⑧ Pseudovirus maturation; ⑨ Pseudovirus release: Mature pseudovirus particles are released from cells via exocytosis and are available for subsequent experiments. (Created with https://BioRender.com/).

Native orthoflaviviruses possess a conserved genome organization, encoding three structural proteins, the capsid protein (C), precursor membrane protein (prM), and envelope protein (E), as well as seven non-structural proteins of NS1, NS2A, NS2B, NS3, NS4A, NS4B, and NS5 [23,24] (Figure 1A,B). Consistent with the native orthoflaviviral genome, viral replicon, a self-replicating viral RNA vector, is typically designed to be derived from the orthoflaviviral non-structural genes but lack viral structural genes. Orthoflavivirus pseudovirus are generally generated using two strategies: either (1) co-transfection of a viral replicon harboring all non-structural protein genes together with an expression vector carrying the C-prM-E structural protein genes or (2) co-transfection of a viral replicon containing the C gene plus all non-structural protein genes with a separate prM-E expression vector [25,26]. In both strategies, viral replicons and structural proteins are expressed in a heterologous expression system via multi-plasmid co-transfection. This process leverages the host cell’s secretory pathway to mimic the assembly and maturation processes of natural orthoflavivirus virions [25,26,27] (Figure 1C,D).

Orthoflavivirus infection is initiated with the binding of envelope protein E to a cognate receptor on the host cell plasma membrane, which induces clathrin-mediated endocytosis. Within the early endosome, protonation of E triggers fusion between the viral and endosomal membranes, releasing the positive-strand genomic RNA into the cytosol. This viral RNA acts as mRNA and is translated by host ribosomes into a single polyprotein [28]. Through co- and post-translational processing by viral and host proteases, the polyprotein is cleaved to produce three structural proteins (C, prM, E) and seven non-structural (NS) proteins. NS proteins remodel endoplasmic reticulum (ER) membranes to form viral replication factories, where RNA synthesis occurs [29,30]. Meanwhile, structural proteins are translocated into the ER lumen. Newly synthesized genomic RNA is packaged by capsid (C) proteins to assemble nucleocapsids, which are subsequently enveloped by prM-E heterodimers to produce immature virions. These immature particles are transported through the secretory pathway to the trans-Golgi network. There, the acidic environment and the host protease furin cleave prM into the mature M protein. This proteolytic cleavage stabilizes the E protein in its mature conformation and renders the particle infectious. Mature virions are finally released by budding at the plasma membrane, thereby initiating a new round of infection (Figure 1E) [31,32].

Similar to natural orthoflavivirus replication, the replicon RNA, serving as the substitute viral genome, is translated by host ribosomes on the rough endoplasmic reticulum (ER), producing a precursor polyprotein encoded by the replicon sequence [33,34]. Proteolytic processing of this precursor polyprotein is primarily mediated by the NS2B-NS3 protease complex [35]. NS2B, a transmembrane cofactor, forms an active complex with the N-terminal serine protease domain of NS3. This complex first undergoes autolysis at the NS2A/NS2B and NS2B/NS3 junctions, followed by cis- and trans-cleavage at the boundaries of other non-structural proteins (including NS1, NS2A, NS3, NS4A, NS4B, and NS5). Concurrently, host signal peptidase localized in the ER membrane completes intramembrane cleavage at the C-terminal site of NS1, releasing the NS1 monomer [35]. To balance replication efficiency and biosafety, viral replicons are engineered to delete structural protein coding regions such as C-prM-E, retaining only non-structural protein regions to block progeny virus assembly. Replicon expression is driven by the strong Human Cytomegalovirus (CMV) promoter ensure efficient transcription [36] (Figure 1F).

Subsequently, the assembly and maturation of pseudoviruses involve multiple host–virus interactions, as follows. During the nucleocapsid formation, the C protein is translated on cytoplasmic ribosomes, then undergoes dimerization and hexamerization to bind replicon RNA, assembling into a nucleocapsid core and completing genome packaging [37]. The interaction between the C protein and intracellular lipid droplets is crucial for efficient viral particle assembly. For specific serotype orthoflaviviruses, such as DENV-2, co-expression of the C protein is essential for pseudovirus production, as it overcomes the low secretion efficiency caused by the strong ER retention signal in the stem-anchor region of the E protein [38] (Figure 1F).

During immature particle assembly, the E protein and its auxiliary prM protein are synthesized on the ER membrane, where they interact to form stable prM-E heterodimers [28,39]. These heterodimers serve as basic structural units, oligomerizing within the ER to assemble into immature particles (~50 nm in diameter) with 60 prM-E trimer spikes on their surface (Figure 1F). For particle maturation, immature particles are transported to the Golgi apparatus via secretory pathway (Figure 1F). Here, the host furin protease cleaves the prM protein, removing the “pr” peptide segment, a molecular chaperone, to generate the mature M protein [28,39]. This cleavage triggers an irreversible conformational rearrangement of the E protein: prM-E heterodimers dissociate, E proteins reassemble into 90 homodimers [40], reshaping the particle surface from a spiky to a smooth, icosahedrally symmetric mature morphology.

Finally, mature pseudoviral particles are released extracellularly via exocytosis. Due to the deletion of structural protein-coding regions in the replicon, these pseudoviruses exhibit only single-round infectivity [38,41].

To facilitate the subsequent detection of viral infection efficiency, a reporter gene, such as a gene encoding green fluorescent protein (GFP) or luciferase (Luc), is typically incorporated into the vector. Successful infection drives reporter gene expression, producing real-time detectable fluorescent or chemiluminescent signals [42]. Promoter selection plays a pivotal role in pseudovirus packaging. Replicon expression vectors commonly use CMV promoter, whose strong transient transcriptional activity enables efficient initiation of non-structural protein expression and genomic RNA replication [36]. In contrast, structural protein expression vectors often employ promoters with stable expression characteristics to maintain continuous, controllable expression of prM/E and other proteins. This design avoids cytotoxicity caused by overexpression [43], and facilitates efficient assembly and secretion of pseudoviral particles, thereby improving pseudovirus packaging efficiency and system safety [44].

### 2.2. Production and Optimization of Orthoflavivirus Pseudovirus

To substantially improve the yield and infectivity of orthoflavivirus pseudoviruses, researchers have implemented a multi-level, systematic optimization strategy covering viral genome modification, host cell selection, and culture system refinement (Table 1).

At the viral genome level, the core objective of viral genome optimization is to enhance the efficiency and fidelity of viral protein expression, thereby laying a foundation for high-quality pseudovirus assembly [45]. Key strategies include genomic sequence engineering, codon optimization, and high-efficiency vector selection [45,46]. In genomic sequence engineering, rational design of viral structural gene sequences, such as truncating non-essential regions of the E protein or optimizing signal peptide sequences, effectively reduces cytotoxicity caused by aberrant protein accumulation, while promoting proper subcellular localization and correct folding of structural proteins (e.g., prM-E heterodimers) [45,47,48]. In addition, as a universal and highly effective approach, codon optimization adapts viral gene sequences to the codon usage bias of host cells, improving translation efficiency and significantly increasing protein expression levels. This strategy is particularly critical for flaviviruses with complex RNA secondary structures, such as DENV-2 and ZIKV [22,46,49]. Additionally, employing engineered expression vectors with enhanced transcriptional activity (e.g., vectors containing modified CMV promoters or enhancer elements) directly boosts the expression of key structural proteins (e.g., E protein and prM protein), thereby increasing pseudovirus production [50]. Furthermore, selecting a viral vector with high compatibility for orthoflaviviral glycoproteins and optimization of its compatibility with the prM-E expression cassette are key to enhancing pseudovirus production efficiency.

Optimization at the host cell and culture level focuses on constructing an optimal microenvironment for pseudovirus packaging, ensuring efficient coordination between viral components and host cellular machinery. Packaging cell line selection is fundamental to pseudovirus production, requiring matching of the cell’s gene expression capacity, secretory pathway efficiency, and assembly compatibility with the specific orthoflavivirus. For example, HEK-293T cells are widely used for broad-spectrum flavivirus pseudovirus packaging due to their high transfection efficiency and robust secretory pathway, while C6/36 cells are preferred for insect-specific flavivirus vectors [11,42,51]. Once an appropriate cell line is selected, the intracellular environment can be further engineered to accelerate the viral protein processing and assembly [26,52]. A key intervention is overexpression of furin, the important host protease responsible for prM cleavage in the trans-Golgi network (Figure 1F). This cleavage releases the pr peptide and generates mature M protein, converting spiky, non-infectious virions into smooth, fully infectious particles. Incomplete processing yields immature or partially mature virions that present alternative antigen conformations and reduced infectivity [53,54,55]. Precise adjustment of culture parameters including medium composition, culture temperature, and pH value. These parameters ensure host cells maintain a healthy metabolic state, which is crucial for sustained high-level production of viral proteins [56]. Finally, adjusting key parameters such as the molar ratio of replicon plasmid to structural protein plasmid, initial cell seeding density and harvest timing, maximizes final yield and infectivity of pseudoviruses [22].

Notably, structural heterogeneity of flavivirus particles is a conserved morphogenetic feature that significantly influences viral immune recognition, host cell tropism, and adaptive capacity [28]. Therefore, during pseudovirus production, strict quality control measures including monitoring particle uniformity via transmission electron microscopy or dynamic light scattering, and structural integrity via Western blot detection of mature M/E proteins, are essential to ensure the reliability and reproducibility of pseudoviruses in subsequent applications.

**Table 1:** Production and optimization strategies of orthoflavivirus pseudovirus.

Optimization	Strategies	Key Points	Reference
Viral Genome Optimization	Genomic sequence engineering	Truncation of non-essential E protein regions (e.g., stem) to reduce steric hindrance and cytotoxicity.	[45,47]
	Codon optimization	Optimized viral gene codons to match the host tRNA pool for maximal translation efficiency, with preservation of cis-acting RNA elements.	[46,48]
	High-efficiency expression vectors	Use of strong promoters and optimized backbones to drive high-level E protein expression.	[50]
Host Cell Regulation	Packaging cell line selection	Selection of packaging cell lines that are compatible with viral gene expression, assembly, and secretion.	[11,42,51,57]
	Targeted cell engineering	Overexpression of key host factors (e.g., furin protease) to ensure precise prM cleavage and particle maturation.	[26,52]
	Culture condition optimization	Optimized culture parameters, including medium composition, temperature, and pH, to enhance production efficiency.	[56]
	Key parameter optimization	Systematic optimization of plasmid ratios, cell seeding density, and harvest time to maximize titer and infectivity.	[22]
Quality Control	Viral particle characterization	Monitoring of particle size, morphology, and integrity via techniques such as negative staining EM and dynamic light scattering to ensure batch-to-batch consistency.	[58,59]

This table summarizes the multi-tiered strategies adopted for the production and optimization of orthoflavivirus pseudoviruses. Systematic optimization of the viral genome and the precise regulation of host cells are crucial to improving pseudovirus yield and infectivity. Furthermore, rigorous quality control for monitoring particle uniformity is emphasized, as the structural heterogeneity of flavivirus particles is a key morphogenetic feature that modulates immune recognition. The integrated application of these strategies is fundamental to generating reliable and high-quality pseudovirus tools, which can be utilized in neutralization assays, vaccine development, and therapeutic antibody research.

## 3. Application of Orthoflavivirus Pseudovirus Technology in Antiviral Research

### 3.1. Screening and Detection of Neutralizing Antibodies

As the core effector molecules of immune defense, neutralizing antibodies (nAbs) can specifically bind to the E protein on the surface of flaviviruses such as DENV and ZIKV. By blocking viral binding and invasion of host cells or inhibiting their replication process, they provide protective immunity [60,61]. The target site of these antibodies is concentrated in domain III of the E protein (EDIII)—the key region determining serotype specificity [62,63].

**Figure 2:**
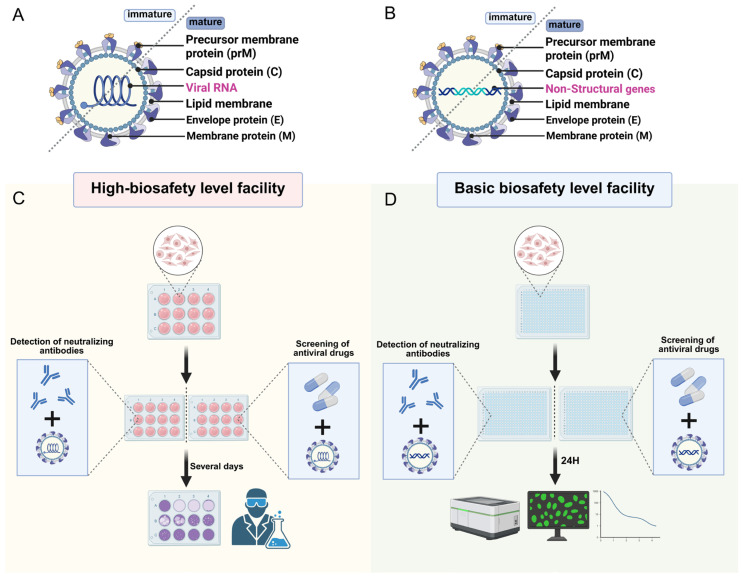
Comparison of workflows for neutralizing antibody detection and antiviral drug screening using live DENV versus DENV pseudovirus. (**A**) Structure of DENV particle; (**B**) Structure of DENV pseudovirus particle; (**C**) Traditional plaque assay workflow. This assay requires high-level biosafety facilities (BSL-3 or above). Live virus is incubated with serum or antibodies, and then inoculated onto monolayer cells. After several days depend on the viral life cycle, viral plaques are counted manually to assess neutralizing activity; (**D**) Pseudovirus-based neutralization assay workflow. This assay can be performed in basic biosafety level facilities. By incorporating a reporter-gene detection system, it enables automated, high-throughput analysis, significantly improving experimental efficiency and safety. It is particularly suitable for large-scale antibody screening and vaccine evaluation. (Created with https://BioRender.com/).

In order to overcome the biosafety limitations in the study of highly pathogenic live viruses, pseudovirus systems have been widely used in the screening of flavivirus neutralizing antibodies [64,65]. Pseudovirus neutralization test (PVNT) simulates the process of virus invasion by using pseudovirus particles packaged with reporter gene, and infects susceptible cells (such as HEK293T) after co-incubation with serum or monoclonal antibody in vitro [66]. If there are effective neutralizing antibodies in the sample, it can inhibit the invasion of pseudovirus and the expression of reporter gene [67]. The neutralization activity can be quantitatively evaluated by detecting the report signal through the high-throughput microplate platform [47,68] (Figure 2). This method not only has high safety and good repeatability, but also supports simultaneous detection of multiple serotypes, which is suitable for large-scale antibody screening and vaccine immunogenicity evaluation.

During the screening of yellow fever virus neutralizing antibodies, multiple experimental methods have been developed and applied at different research stages, each with its own applicability and limitations (Table 2). For instance, PVNT has become a crucial method for early antibody screening and large-scale neutralization capacity assessment due to its high safety and high-throughput characteristics. This method not only significantly enhances detection efficiency—exceeding the traditional plaque reduction neutralization test (PRNT) by over 50-fold [47,68]—but also reduces the coefficient of variation (CV) in inter-laboratory data to below 15% due to its excellent reproducibility, markedly outperforming the 30% fluctuation observed with PRNT [61,68]. As the gold standard for evaluating the activity of neutralizing antibodies, plaque reduction neutralization test (PRNT) can fully reflect the neutralization ability in the viral replication cycle, but it is often used for confirmatory testing due to its complicated operation, high biosafety requirements and limited throughput [69]. Other methods like the micro neutralization test (MNT) strike a balance between throughput and safety, making it suitable for medium-scale screening [69,70].

Therefore, integrating these techniques into a tiered pipeline from high-throughput primary screens to confirmatory assays, which represents the most effective approach for the accurate identification of orthoflavivirus-neutralizing antibodies.

Notably, the maturation status of pseudovirus particles is a critical variable that must be considered when interpreting neutralization assay results. The extent of prM cleavage determines the conformation of the E protein displayed on viral particle surface, and the E protein serves as the primary target of neutralizing antibodies [55]. In immature virus particles, the E protein typically forms a complex with uncleaved prM, resulting in partial masking or a suboptimal conformation. This not only restricts the recognition of neutralizing antibodies but may also induce the production of non-neutralizing antibodies, thereby increasing the risk of immune escape. Mature E proteins are exposed on the particle surface as dimers, enabling neutralizing antibodies to efficiently bind to key regions such as the fusion loop and inhibit viral entry [47,55]. Therefore, strict quality control should be implemented both in the preliminary evaluation of neutralizing antibodies using pseudoviruses and in subsequent PRNT validation with live viruses to minimize result deviations caused by the maturation status of viral particles.

In orthoflavivirus vaccine development research, pseudoviruses serve as an efficient tool for evaluating vaccine efficacy and quality based on a reliable neutralizing antibody assay [73]. The E and prM proteins expressed by pseudoviruses closely mimic native viral conformation and retain immunogenicity, making them ideal neutralizing antigens for antibody screening and functional analysis. Currently, pseudovirus-based neutralization assays are now widely used in both preclinical and clinical development of flavivirus vaccines, such as for JEV and YFV, to measure neutralizing antibody titers in immune sera [74,75,76,77].

Beyond immunogenicity assessment, pseudovirus technology provides a versatile platform for mechanistic studies and vaccine design. It enables the construction of chimeric viruses to deeply explore the viral genomic functions and epitope mechanisms, providing theoretical and experimental basis for the novel vaccine design. Simultaneously, it supports precise quantification of vaccine-induced neutralization and serves as a key platform for investigating antibody-dependent enhancement effects [78,79,80]. Although approved specific vaccines are still absence for most flaviviruses so far, the application of pseudovirus technology has significantly accelerated preclinical evaluation, shortened development timelines from laboratory research to clinical translation [11,81], and provided critical technical support for the prevention and control of flavivirus diseases.

### 3.2. Screening and Evaluation of Antiviral Drugs

By simulating key steps in the orthoflavivirus life cycle, pseudovirus technology offers a high-throughput screening platform for antiviral drug development. Particularly, pseudoviruses mimic the processes of viral binding and membrane fusion, enabling systematic screening of viral entry inhibitors and evaluation of drug cross-neutralization against different serotypes or flavivirus species under routine biosafety conditions (Figure 2). In DENV research, pseudovirus technology has confirmed that DENV infection can be blocked by viral NS5 polymerase inhibitor, but not by host α-glucosidase inhibitors [82]. In Zika virus (ZIKV) research, pseudovirus technology has demonstrated strengths in screening antiviral compound specific to viral entry. It has verified that the small-molecule compound AMS (a thiol-conjugating reagent) effectively blocks live virus entry [83]. Moreover, it has shown that Arbidol inhibits ZIKV infection by blocking virus–host membrane fusion during early viral entry [84]. Even in broad-spectrum antiviral screening, pseudovirus technology has achieved remarkable progress. In West Nile virus research, pseudovirus technology has been utilized to evaluate the inhibitory effects of drugs against multiple orthopoxviruses, significantly improving screening efficiency [85]. Overall, pseudovirus technology provides a safe, straightforward, and versatile strategy for discovering inhibitory antibodies, peptides, or small molecules against orthoflaviviruses.

Pseudovirus system was also been successfully used to evaluate broad-spectrum inhibitors targeting flavivirus NS5 protein [86], and to verify the feasibility of claudin family proteins as anti-flavivirus drug targets [87]. Notably, the mitochondrial-localized CMPK2 protein exerts antiviral functions through its N-terminal domain. Pseudovirus experiments have confirmed that it exhibits inhibitory effects on multiple flaviviruses, demonstrating its potential as a pan-yellow virus inhibitor [88]. In the experimental verification, compound 16a showed potent inhibition against DENV-2 (EC_50_ = 1.4 μM) and ZIKV (EC_50_ = 2.4 μM) [89]. Another study reported that compound 19 containing a pyrazine structure has broad-spectrum anti-flavivirus activity, while compounds containing thiophene structure exhibit selective inhibition against JEV-SA14 and YFV-17D [90].

These research advances indicate that the flavivirus pseudovirus system has become an indispensable tool in antiviral drug development, and exhibits unique advantages especially in the screening of antiviral compounds targeting the early-stage of viral replication and mechanism research [71,91]. By integrating computational prediction and experimental verification, this technology platform is accelerating the discovery process of broad-spectrum anti-flavivirus drugs.

## 4. Latest Advances and Challenges

### 4.1. Latest Advances

Pseudovirus technology significantly reduces biosafety risk by enabling only a single round of infectivity, which prevents viral spread beyond initial entry. This feature allows researchers to safely assess neutralizing antibody activity, screen antiviral compounds, and characterize the antigenicity of specific viral strains. In contrast, live virus system more accurately replicates natural infection and thus offer higher clinical relevance for antiviral drug and vaccine development [78,79]. However, they require high biosafety level facilities and carry the inherent risk of viral mutation during experimentation [79]. These constraints substantially increase the difficulty of conducting early-stage antiviral research. Consequently, pseudovirus technology provides a more practical and biosafety-friendly alternative, particularly in standard laboratory environments.

Pseudovirus technology substantially shortens the testing cycle and greatly reduces the time required for research and screening of antiviral compounds and vaccines. It transforms traditionally labor-intensive neutralization assays and drug screening into efficient, scalable, and standardized workflows [74,92]. This approach allows researchers to assess the neutralizing or inhibitory effects of antibodies or antiviral molecules on viral infection within 24 h post infection, overcoming the time constraints associated with live-virus assays that depend on a complete viral replication cycle [92]. Moreover, the fluorescent reporter genes encoded in pseudoviruses enable seamless integration with high-throughput automated fluorescence analysis systems. This integration establishes an efficient and automated platform for rapid viral identification and diagnosis, large-scale serological surveillance, and high-throughput screening of antiviral compounds and neutralizing antibodies [93].

Pseudovirus technology retains the ability to mediate viral entry and incorporate viral replicon encoding non-structural proteins. This design not only enables its use not only in studying viral invasion mechanisms, receptor recognition, and neutralization assays [18], but also in recapitulate key steps of live viral replication, such as glycosylation-dependent endoplasmic reticulum homeostasis and viral replication compartments formation, through NS1 protein [94]. Compared with virus-like particles (VLPs), pseudovirus offer a more dynamic model for investigating virus–host interactions within cells. VLPs, are composed of self-assembled viral structural proteins, closely resemble native virions in morphology. Owing to their favorable modifiability, carrier properties, and lacking viral nucleic acid, they present reduced biosafety risks and can effectively elicit robust humoral and cellular immune responses. These characteristics make VLPs particularly valuable for vaccine design and antigen characterization [80,81,95], with additional applications in targeted delivery [96,97,98]. Nevertheless, VLPs still faces challenges such as limited immunogenicity and variable protein self-assembly efficiency [15,99] (Table 3).

In summary, pseudoviruses, VLPs, and live viruses form a complementary toolkit in modern antiviral research. Integrating their respective strengths can advance both foundational understanding and translational development, ultimately accelerating the discovery of antiviral drugs and vaccines and improving preparedness against emerging viral caused public health threats.

### 4.2. Limitations

**Figure 3:**
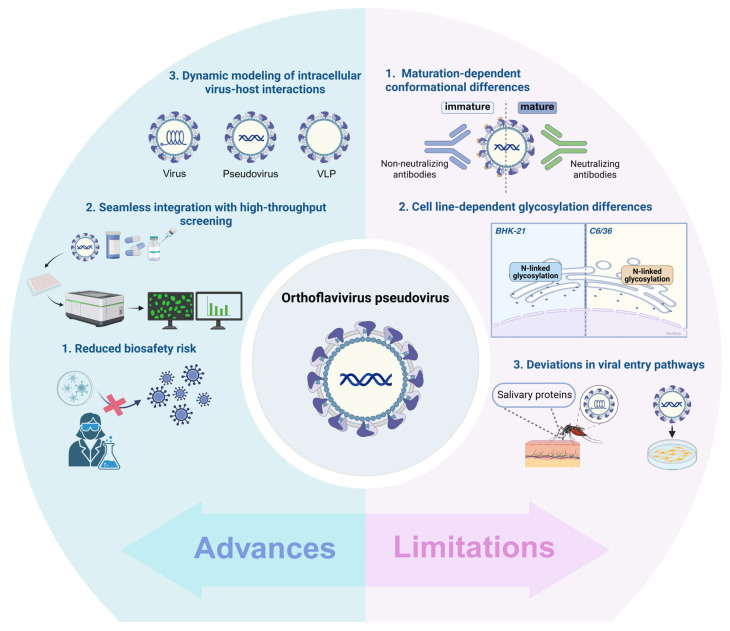
A schematic diagram of the frontier applications and challenges of orthoflavivirus pseudovirus technology. The left panel highlights important advances: ① Reduced biosafety risk: Pseudovirus technology allows safe study of orthoflavivirus under basic biosafety level facility, significantly reducing the risk associated with handling live viruses; ② Seamless integration with high-throughput screening: Pseudovirus technology seamlessly integrates with high-throughput screening processes, enabling automation and rapid screening of extensive antibody/drug libraries; ③ Dynamic modeling of intracellular virus–host interactions: Pseudovirus technology retains the capability to mediate viral entry and incorporate viral replicon encoding non-structural proteins. The right panel highlights notable limitations: ① Maturation-dependent conformational differences: Immature pseudoviruses may lack mature conformations, which could potentially skew immune evaluations and affect the accuracy of research outcomes; ② Cell line-dependent glycosylation differences: Glycosylation patterns in producer cells differ from those in target cells, which might impact antibody recognition; ③ Deviations in viral entry pathways: Pseudovirus technology may not fully recapitulate the complex, multi-step entry process of live viruses associated with salivary proteins. (Created with https://BioRender.com/).

Although pseudovirus technology has shown great potential in flavivirus research, it still has several technical limitations that warrant attention (Figure 3).

First of all, heterologous viral glycoproteins displayed on immature pseudoviruses may not fully replicate the distribution, conformation, and density on native live viruses [50], which may alter antibody or drug recognition of E protein epitopes and lead to pseudovirus-based neutralization results that do not fully reflect immune responses in genuine infections [15,50]. Moreover, viral particles undergo dynamic conformational changes during maturation and entry, which immature pseudovirus may not fully recapitulate these continuous conformational dynamics. Therefore, the evaluation of certain neutralizing antibodies or inhibitors may be influenced by the intermediate conformational states [29,105]. Additionally, uneven antigen density on the pseudovirus surface may cause “epitope masking” phenomenon, where high-affinity antibodies preferentially bind to exposed epitopes and physically hinder B cell recognition of adjacent epitopes, potentially skewing immune evaluation [106].

Secondly, glycosylation differences associated with production cell lines pose another limitation. The N-linked glycosylation of flavivirus envelope proteins (such as the E protein of ZIKV and DENV) is critical for proper protein folding, immune evasion, and sensitivity to antibody neutralization [107,108]. If pseudoviruses are produced in heterologous cell lines, where glycosylation enzymes and modification patterns often differ substantially from those of the virus’s natural target cells (e.g., human or mosquito cells) [109,110]. This can lead to alterations in the glycoforms at key glycosylation sites of the E protein, thereby affecting relevant biological functions and assay outcomes.

Thirdly, pseudoviruses may exhibit deviations in viral entry pathways. Natural infection by orthoflavivirus typically triggers viral entry pathways (e.g., via the TLR2 pathway) with the assistance of vector salivary proteins, which modulates local host immunity and facilitates infection [111]. Such complex biological contextual factors are generally not recapitulated in in vitro pseudovirus infection assays.

### 4.3. Key Challenges

While pseudovirus technology is widely applied, standardization across laboratories remains a significant challenge. Currently, international standards for anti-SARS-CoV-2 immunoglobulin (e.g., WHO NIBSC 20/136) have been established to calibrate neutralization assays and provide critical guidelines for pseudovirus-based experiments [112,113]. However, the absence of specific national standards for pseudovirus detection limits broader consistency. Establishing such standards would greatly enhance data comparability and reproducibility across diverse research and clinical settings, thereby supporting more reliable application in vaccine evaluation and drug development. It is a direction that deserves further investigation. Furthermore, batch-to-batch variability in pseudovirus production poses a challenge to quality control. This inconsistency primarily stems from three factors: (1) the cis-acting RNA elements in the viral genome may not retain their native conformation in pseudovirus system, thereby reducing packaging efficiency [29]; (2) fluctuations in the expression of key host factors such as the endoplasmic reticulum remodeling protein TMEM41B can lead to unstable pseudovirus yields [30,114]; (3) the maturation of flaviviruses depends on the precise cleavage of the prM protein; however, the efficiency of protease processing in pseudovirus systems is variable, which further impacts pseudovirus yield and infectivity [54,115]. Given these limitations, caution is necessary when interpreting pseudovirus experimental results. It is highly recommended to verify the determination results of pseudoviruses with experiments based on live viruses, and to employ a combination of complementary techniques for comprehensive evaluation [12,116].

## 5. Conclusions

As an important biomedical research tool, orthoflavivirus pseudovirus technology has achieved remarkable contributions to antiviral research. In basic research, it has been instrumental in elucidating the structural mechanisms of virus–host interaction, viral maturation, and identifying conserved targets, thereby providing a theoretical foundation for the design of broad-spectrum antiviral drugs and vaccines [117,118]. At the application level, pseudovirus systems facilitate the clarification of critical immune mechanisms (e.g., antibody-mediated “epitope masking”) and have promoted the development of novel antiviral strategies, including defective interfering particles (DIPs) and engineered extracellular vesicle (EV) delivery systems [119,120]. Crucially, it provides a safer alternative model for studying highly pathogenic viruses under standard laboratory conditions. However, it should be noted that work with pseudoviruses still requires appropriate biosafety facilities and strict compliance with operational protocols [47,121].

Despite the widespread application of pseudovirus technology, further efforts are still required to advance its standardization. Factors such as different production cell lines and plasmid construction strategy may lead to batch-to-batch variations in the production of pseudoviruses, while the lack of standardized reference materials also limits the quantitative comparison between different research [47,122]. In addition, preliminary screening results based on pseudoviruses must be validated through in vitro and in vivo experiments using live viruses [123]. Therefore, to scientifically guide the application of this technology in antiviral research, a tiered research strategy is recommended: in the early stages of drug or antibody development, high-throughput screening is recommended for pseudoviruses; in subsequent critical stages, functional validation via live virus experiments in in vitro and in vivo models must be performed to ensure the reliability and translational value of the results.

Looking forward to the future, several strategic directions will be critical for advancing this technology. These include developing novel targeted nano-delivery systems to improve drug efficacy and bioavailability, integrating advanced technologies such as cryo-electron microscopy to resolve key steps in viral entry, and constructing comprehensive pseudovirus libraries covering circulating strains and variants to keep pace with viral evolution [120,124,125,126]. In addition, innovative strategies such as nucleic acid hydrolysis-targeting chimeras (NATAC) represent a promising avenue for developing broad-spectrum antivirals [127]. Furthermore, establishing a standardized and quality-controlled framework is essential to guarantee the quality of pseudoviruses. This framework should encompass the development of widely recognized reference pseudovirus libraries, standardized titration methodologies, and unified reporting guidelines, all of which will significantly enhance the comparability and reproducibility of experimental data. Achieving these goals will require deeper interdisciplinary collaboration across virology, nanotechnology, structural biology, and computational science focusing on core challenges such as targeted drug delivery, real-time monitoring of viral escape mutations, and the design of broad-spectrum antiviral strategies [117,120,125,126]. Through sustained innovation and cross-disciplinary synergy, flavivirus pseudovirus technology will continue to serve as a central research engine, providing robust and sustained support for in-depth elucidation of viral pathogenic mechanisms and accelerated development of efficient antiviral interventions.

## Figures and Tables

**Table 2:** Comparison of common neutralization assays for orthoflaviviruses.

Methods	Strengths	Limitations	Application	Reference
Pseudovirus Neutralization Test (PVNT)	High safety; Flexibility; High throughput; High sensitivity.	Limited viral life cycle mimicry; Specific host cell requirements.	Large-scale profiling of vaccine-induced serum responses; High-throughput screening for neutralizing antibody therapeutics; Simultaneous analysis of multiple viral serotypes.	[12,50,71]
Plaque Reduction Neutralization Test (PRNT)	The gold standard; Reflects complete neutralization activity.	High-level biosafety facilities requirements; Labor-intensive and prolonged operational workflows; Limited throughput.	Confirmatory testing; Preclinical evaluation; Seroepidemiology.	[69]
Micro-Neutralization Test (MNT)	Moderate flux; Wide range of applications.	Mandatory use of live viruses under high biosafety levels; there may be subjective bias.	Seroepidemiological survey; Vaccine immunogenicity evaluation.	[70,72]

This table provides a comparative overview of standard assays for detecting orthoflavivirus neutralizing antibodies, including Pseudovirus Neutralization Test (PVNT), Plaque Reduction Neutralization Test (PRNT), Micro-Neutralization Test (MNT). For each technique, the strengths, limitations, and typical applications are outlined. The summary is intended to guide the selection of optimal strategies for various applications, from antibody discovery to vaccine immunogenicity assessment, by balancing key factors like safety, throughput, physiological relevance, and practical implementation.

**Table 3:** The comparison of pseudoviruses and virus-like particles (VLPs).

Feature	Pseudoviruses	Virus-Like Particles (VLPs)	Reference
Structure	Orthoflavivirus structural protein and replication-defective genome.	Orthoflavivirus structural protein without viral genome.	[12,100]
Replication	Single-round infection.	Non-replicative.	[11,101]
Strength	Simulates the early stages of viral infection, High throughput, Studying viral variants.	Reduced biosafety risk, Strong immunogenicity, Carrier properties.	[12,102]
Limitation	Potential safety issues associated with viral defective genomes, Batch-to-batch production standardization.	Defective in replication, Low self-assembly efficiency.	[12,103,104]

This table provides a comparative summary of pseudoviruses and VLPs as distinct biotechnology tools. Their core designs differ: pseudoviruses serve as single-round infection models for mechanistic invasion studies, while VLPs function as non-infectious immunogenic scaffolds. The comparative framework encompasses key parameters including safety characteristics, replication capacity, aiming to clarify the inherent strengths and limitations of both platforms.

## Data Availability

No new data were created or analyzed in this study. Data sharing is not applicable to this article.
